# In Vivo Anti-Inflammatory Effect, Antioxidant Activity, and Polyphenolic Content of Extracts from *Capsicum chinense* By-Products

**DOI:** 10.3390/molecules27041323

**Published:** 2022-02-16

**Authors:** Lilian Dolores Chel-Guerrero, Gabriela Castañeda-Corral, Misael López-Castillo, Matteo Scampicchio, Ksenia Morozova, Julio Enrique Oney-Montalvo, Giovanna Ferrentino, Juan José Acevedo-Fernández, Ingrid Mayanín Rodríguez-Buenfil

**Affiliations:** 1 Centro de Investigación y Asistencia en Tecnología y Diseño del Estado de Jalisco A. C. Subsede Sureste, Tablaje 31264 km, 5.5 Carretera Sierra Papacal-Chuburna Puerto, Parque Científico Tecnológico de Yucatán, Mérida C.P. 97302, Mexico; ldch.guerrero7@gmail.com (L.D.C.-G.); juoney_al@ciatej.edu.mx (J.E.O.-M.); 2 Universidad Autónoma del Estado de Morelos, Av. Universidad No. 1001, Col Chamilpa, Cuernavaca C.P. 62209, Mexico; gabriela.castaneda@uaem.mx (G.C.-C.); misael.pez@gmail.com (M.L.-C.); 3 Faculty of Science and Technology, Free University of Bozen-Bolzano, Piazza Università 5, 39100 Bolzano, Italy; matteo.scampicchio@unibz.it (M.S.); ksenia.morozova@unibz.it (K.M.)

**Keywords:** *C. chinense* by-products anti-inflammatory activity, antioxidant activity, polyphenolic content, extraction technologies, type of soil

## Abstract

By-products of *Capsicum chinense* Jacq., var Jaguar could be a source of bioactive compounds. Therefore, we evaluated the anti-inflammatory effect, antioxidant activity, and their relationship with the polyphenol content of extracts of habanero pepper by-products obtained from plants grown on black or red soils of Yucatán, Mexico. Moreover, the impact of the type of extraction on their activities was evaluated. The dry by-product extracts were obtained by maceration (ME), Soxhlet (SOX), and supercritical fluid extraction (SFE). Afterward, the in vivo anti-inflammatory effect (TPA-induced ear inflammation) and the in vitro antioxidant activity (ABTS) were evaluated. Finally, the polyphenolic content was quantified by Ultra-Performance Liquid Chromatography (UPLC), and its correlation with both bioactivities was analyzed. The results showed that the SFE extract of stems of plants grown on red soil yielded the highest anti-inflammatory effect (66.1 ± 3.1%), while the extracts obtained by ME and SOX had the highest antioxidant activity (2.80 ± 0.0052 mM Trolox equivalent) and polyphenol content (3280 ± 15.59 mg·100 g^−1^ dry basis), respectively. A negative correlation between the anti-inflammatory effect, the antioxidant activity, and the polyphenolic content was found. Overall, the present study proposed *C. chinense* by-products as a valuable source of compounds with anti-inflammatory effect and antioxidant activity.

## 1. Introduction

Nowadays, numerous anti-inflammatory agents are used to relieve the aches and pain of everyday life, as well as for the treatment of the symptoms of many diseases, such as rheumatoid arthritis, osteoarthritis, and psoriasis. These include drugs such as ibuprofen, indomethacin, diclofenac, and naproxen. However, despite its excellent anti-inflammatory effectiveness, the severe associated side effects (e.g., gastrointestinal ulceration, perforation, obstruction, and bleeding) very often limit their therapeutic use, especially in chronic treatments [1,2]. Accordingly, the development of novel, effective and safe anti-inflammatory agents has been a continuously desirable goal. Consequently, many scientific investigations have been performed over the years searching for anti-inflammatory compounds from natural sources. There is a consensus on the beneficial effects on human wellness of a diet rich in fruits and vegetables, as sources of polyphenolic compounds, such as quercetin, apigenin, hesperidin, and luteolin [3]. These polyphenolic compounds have demonstrated several biological properties, which result in the modulation of the homeostasis of key cellular processes, such as the metabolism, the energy balance, the redox equilibrium, the cell signaling, the inflammatory response, and the control of oxidative stress [3,4,5,6].

Currently, there is a growing interest in the study of antioxidant and anti-inflammatory activities of vegetable and fruit by-products. Published findings have shown that the non-edible parts of vegetables and fruits often have a higher content of functional compounds than the edible parts. That is the case of peels of lemons, oranges, and grapefruit, which presented polyphenolic content 15% higher than the peeled fruits [7,8]. Polyphenols are also found in fruits and by-products of *Capsicum chinense* Jacq. (Habanero pepper) [9], a very aromatic variety of peppers that has its origin in Mexico and South America. It is claimed to be the hottest chili pepper in the world. The fruits and leaves of the habanero are used in traditional medicine for the treatment of asthma, cough, sore throat, tooth pain, muscle pain, gastrointestinal diseases, and arthritis [10,11]. Additionally, some researchers have proved that the bioactive extracts and their bioactive compounds act by blocking two major signaling pathways: (1) the nuclear factor kappa B (NF-κB); and (2) the mitogen-activated protein kinases (MAPKs), both involved in the production of various pro-inflammatory mediators [5].

In 2010, the habanero pepper was granted with Designation of Origin by the Mexican Institute of Industrial Property (IMPI) [12], and nowadays, it is the main horticultural species commercially exploited in the Southeastern of Mexico with around 358.37 ha cultivated (5049 T). In this farming zone, there are two types of characteristic leptosols where the habanero is cultivated, “*Káankab luum*” or red soil and “*Box luúm*” or black soil [13,14]. The difference between the red and black soils is due to their organic and inorganic composition of available nutrients. Both have different content of calcium carbonates, organic matter, nitrogen, and phosphorus, being the black soil the one with the highest contents. The soil composition may be one of the factors responsible for the development and the biosynthesis of polyphenols in the habanero pepper affecting its antioxidant activity. As an example, the higher content of organic matter and nitrogen content affects the activity of the phenylalanine ammonia-lyase (PAL), an enzyme involved in the biosynthesis of polyphenols in the species of the genus *Capsicum* [15].

During the cultivation and industrialization process, many habanero by-products are disposed of. These include approximately 7.9 million plants and 155.3 million peduncles per year (considering 22,000 plants·ha^−1^ on average with the 20% of the fruits industrialized corresponding to 6.5 g of average weight per fruit) [9,11,12]. However, some reports have shown that leaves, peduncles, and stems contain significant amounts of polyphenolic compounds. Specifically, it has been reported that the stems extracts have an abundance of flavonoids like protocatechuic and chlorogenic acid [9,10,11,12,13,14,15,16]. Moreover, a recent study showed that extracts of *C. chinense* (red pepper) cultivated in Nigeria presented in vitro anti-inflammatory activity. The authors found that the hydromethanolic extracts of seeds, fruits, and leaves exhibited significant membrane stabilization properties between 71% and 68%, comparable to those displayed by the positive control Diclofenac (80%) in the Human Red Blood Cell membrane stabilization assay. In the lysis model induced by hypotonic solution and heat, the leaves extract showed less protection (58%) against protein denaturation than Diclofenac (80%) [17].

However, the extraction technology applied for the recovery of such compounds may greatly affect their bioactivity and bioavailability. Nowadays, there is a growing interest in replacing conventional solvent extraction (CSE) methods (e.g., percolation, maceration, and Soxhlet techniques) with green technologies, since they require less time for extraction, no degradation of target compounds, and no consumption of hazardous solvents. Some of these technologies include pressurized liquids, supercritical fluid, microwave-assisted, and ultrasound-assisted extraction. These methods and the use of CO_2_, H_2_O, or NH_3_ as non-polluting and easy-to-remove solvents may improve the product quality by fastening the extraction. However, although conventional solvent extraction technologies have several drawbacks, several studies have demonstrated that they show good recovery of polyphenols obtaining biologically active extracts [18,19,20].

To the best of our knowledge, there are no studies published so far comparing the anti-inflammatory and the antioxidant activities and their relationship with the polyphenolic content of extracts of *C. chinense J*., var Jaguar by-products, obtained by green and conventional extraction methods.

Thus, this study aims to evaluate the in vivo anti-inflammatory effect, the in vitro antioxidant activity, and their relationship with the polyphenolic content of extracts of habanero pepper by-products, taking into consideration whether they were grown on black or red Yucatan soil, and to determine the impact of supercritical fluid extraction versus maceration and Soxhlet on their bioactivities.

## 2. Results and Discussion

In this research, the extracts of leaves, peduncles, and stems of habanero pepper from plants grown on black and red soils of Yucatán were obtained first by maceration. Next, Soxhlet and supercritical fluids extraction were applied to recover bioactive compounds from stems of plants grown on both types of soils and from leaves of plants grown on black soils [9]. In both cases, the in vivo anti-inflammatory activity, the antioxidant activity, and the quantification of polyphenols by UPLC were determined and compared. Finally, the dose–response curves of the anti-inflammatory effect of the most active extracts were obtained.

### 2.1. Anti-Inflammatory Effect of Extracts Obtained by Maceration

Inflammation is a protective or healing response to infection and tissue injury. Its resolution is central to human health. The anti-inflammatory treatment goal is to reduce or eliminate swelling and pain. Hence, the development of more effective and less toxic drugs continues to be a challenge for researchers in order to provide new therapeutic avenues for the treatment of these diseases [21].

**Figure 1:**
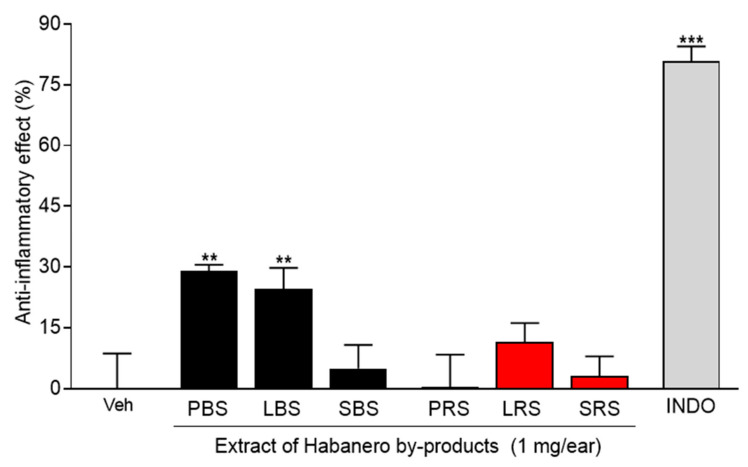
Anti-inflammatory effect of the methanolic extracts obtained by maceration from peduncles (P), leaves (L), and stems (S) of plants of *C. chinense* grown in black (BS) or red soils (RS) of Yucatán in the mouse model of TPA-induced ear edema. Each bar represents the mean ± SEM (n = 6–8 per group). ** *p* < 0.01, *** *p* < 0.001, different from the vehicle (Veh) group by one-way ANOVA followed by Dunnett’s test. INDO: Indomethacin.

In this study, the anti-inflammatory effect of extracts of different by-products of habanero pepper such as leaves, peduncles, and stems of plants grown on black or red soil was evaluated in a mouse model of inflammation. TPA (12-O-tetradecanoylphorbol-13-acetate)-induced ear edema has been widely used as a screen model to identify compounds with anti-inflammatory activity. Firstly, a preliminary test was performed to assess the effect of extracts obtained by maceration with methanol at the dose of 1 mg·ear^−1^. The results showed that only the extracts of peduncles and leaves of plants grown on the black soil of Yucatán exhibited a significant anti-inflammatory effect, corresponding to 28.91 ± 1.61% and 24.60 ± 5.14%, respectively. One-way ANOVA analysis indicated no statistically significant differences between the effects exhibited by both extracts (Figure 1). However, peduncles of plants grown on red soil reported the lowest anti-inflammatory effect. This was probably associated with the different bioactive compounds with anti-inflammatory activity detected in the sample. They could be present at higher or lower concentrations depending on the solubility and affinity for the solvent used during the extraction. On the other hand, the type of by-products and the soil were factors with a statistically significant effect on the anti-inflammatory effect at a 95% confidence level. However, the interaction of both factors did not present a statistically significant effect on the response variable. 

The results agreed with the study of Ukwani and Hassan [17], who reported that the hydromethanolic extract of *C. chinense* leaves cultivated in Nigeria, at a concentration of 400 μg·mL^−1^, exhibited in vitro significant anti-inflammatory activity comparable to the standard drug Diclofenac in the Human Red Blood Cell membrane stabilization assay.

In this study, the observed difference in the anti-inflammatory effect could be linked to the type of by-products, as different metabolites are stored in different plants organs (e.g., alkaloids in the leaf surface, flavonoids and terpenoids in roots and leaf) [22]. Moreover, the soil composition also played an important role in the profile and content of secondary metabolites in plants affecting their synthesis and accumulation. The black soil presented a higher content of calcium carbonates, organic matter, nitrogen, and phosphorus compared to the red soil. The relationship of polyphenol and terpenoids content with nitrogen is particularly strong. Studies indicated that these type of metabolites showed a strong anti-inflammatory activity [15,23,24,25,26]. Additionally, the results of the present study confirmed the higher anti-inflammatory activity of extracts obtained from plants grown on black soil.

### 2.2. Antioxidant Activity of Extracts Obtained by Maceration

In the present study, the antioxidant activity of the extracts of habanero by-products obtained by maceration was assessed by ABTS. The results showed that the extracts of leaves, peduncles, and stems of plants grown on black or red soil displayed antioxidant activity in a range between 0.74 and 2.57 mM Trolox equivalent, with the extracts of peduncles and stems being more active. In contrast, the extracts of leaves grown on black or red soil showed the lowest antioxidant activity (Table 1). The type of by-product, the type of soil, and the interaction of both factors exerted a statistically significant effect on the antioxidant activity with a 95.0% confidence level. One-way ANOVA analysis showed that the stems of plants grown on red soil had the highest antioxidant activity (2.80 ± 0.01 mmol Trolox equivalent per mg dry extract (DE)).

Rodriguez Buenfil et al. [9] reported that the methanolic extracts of peduncles and stems of *C. chinense*, grown on black soil and red soil of Yucatán, presented higher antioxidant activity (determined by the DPPH assay) than the leaves of both types of soil. This finding agreed with the results of the present study, obtained using the ABTS method. They also reported that the samples displaying the highest percentage of inhibition were the stems (41.15 ± 0.22%) and the peduncles (49.55 ± 0.16%) grown on black and red soils, respectively. In this study, only the stems of plants grown on red or black soils exhibited the highest antioxidant activity. This result was probably affected by the different extraction method applied for the recovery of the compounds as maceration resulted less efficient in the extraction from peduncles. It is well noted, in fact, that ultrasound assisted extraction can enhance and facilitate the extraction and release of compounds from plants.

### 2.3. Polyphenolic Content of Extracts Obtained by Maceration

Polyphenols are natural compounds found abundantly in plants, including habanero peppers. In this study, the polyphenol content of the different extracts of habanero by-products obtained by maceration was quantified by UPLC. The results showed that peduncles, leaves, and stem extracts of *C. chinense* grown on red soil reported a greater content of polyphenols as compared to the extracts of peduncles, leaves, and stems of plants grown on black soil (Table 2). The extract of peduncles of plants grown on red soil showed the highest total polyphenol content (2638 ± 87.44 mg·100 g^−1^ Dry basis (DB)). Individually, 11 polyphenols were detected in all the samples at concentrations ranging from 26.53 ± 0.64 to 265.13 ± 1.63 mg 100 g^−1^ of DB. The polyphenols found in higher amounts were quercetin, luteolin, myricetin, and chlorogenic acid. The extract of stems of *C. chinense* grown on red soil showed the highest content of quercetin and luteolin (562.10 ± 1.47 mg·100 g^−1^ DB). The extracts from leaves of *C. chinense* grown on red soil exhibited the highest content of myricetin (489.33 ± 16.57 mg·100 g^−1^ DB), while the extracts from leaves of *C. chinense* grown on both types of soil showed the highest content of chlorogenic acid (308.53 ± 1.53 and 311.13 ± 0.50 mg·100 g^−1^ DB, black and red soil, respectively). Catechin and diosmetin were only identified in the peduncles and leaves, respectively, of plants grown on red soil. Kaempferol was identified in peduncles and leaves of plants grown on both types of soils, and naringenin in peduncles of plants grown on both types of soils and leaves of plants grown on red soil. Gallic acid, protocatechuic acid, and apigenin were not detected in the analyzed samples. 

In a previous study, it was reported that the extracts obtained by ultrasound treatment of leaves and peduncles of plants grown on black soil showed a higher polyphenol content than the extracts of leaves and peduncles of plants grown on red soil. The results were justified by considering the differences related to the physicochemical composition of black soil rich in nitrogen and organic matter [15,27]. In this study, the same raw material and the same types of soil were used. However, a different extraction method (maceration vs. ultrasound) and solid–liquid ratio (2 mg·mL^−1^ vs. 1 mg·mL^−1^) was applied. These two parameters could explain the different results obtained as both factors could affect the extraction efficiency and the solubility and selectivity of the different compounds [28]. Indeed, a higher solvent–liquid ratio avoided the saturation phenomenon, thus allowing a continuous and complete release and diffusion of the compounds from the plants to the solvent.

### 2.4. Correlation between the Anti-Inflammatory Effect or the Antioxidant Activity with the Polyphenolic Content of the Extracts Obtained by Maceration

The correlation between the anti-inflammatory effect and the antioxidant activity or the polyphenol content of the extracts obtained by maceration was analyzed (Table 3). Regarding the antioxidant activity, a positive correlation (r = 0.97, *p* < 0.05 and r = 0.92, *p* < 0.05) was found between cinnamic acid, rutin and antioxidant activity. On the other hand, a negative correlation (r = −0.83, −0.72, *p* < 0.05) was found between chlorogenic acid and myricetin content and the antioxidant activity, respectively. Overall, a negative correlation was found between the antioxidant activity and the anti-inflammatory effect (r = −0.51, *p* < 0.05). Finally, a moderate positive correlation was observed between the kaempferol content and the anti-inflammatory effect (r = 0.54, *p* < 0.05).

The Pearson’s correlation coefficients showed that cinnamic acid and rutin were the main compounds responsible for the antioxidant activity of the samples. The result was consistent with reports showing that both cinnamic acid and rutin reported a strong antioxidant activity [29,30]. Additionally, these results suggested that the antioxidant activity was not the main factor responsible for the anti-inflammatory effect. The correlation with kaempferol content, a polyphenol with anti-inflammatory activity through the NF-κB, IκK-NEMO association domain and IκK [31], suggested the involvement of other mechanisms. However, additional experiments are needed to determine which polyphenols are involved in the observed anti-inflammatory effect.

### 2.5. Comparison of the Anti-Inflammatory Effect of Extracts Obtained by Maceration (ME), Soxhlet (SOX), and Supercritical Fluid Extraction (SFE)

**Figure 2:**
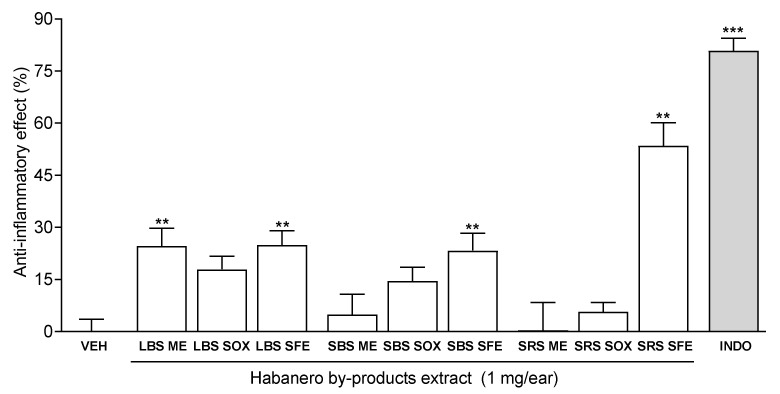
Anti-inflammatory effect of habanero pepper by-product extracts obtained by maceration (ME), Soxhlet (SOX) and supercritical fluid extraction (SFE). Each bar represents the mean ± SEM (n = 6–8 per group). *** *p* < 0.05, ** *p* < 0.01, different from the group treated with the vehicle (VEH), by one-way ANOVA followed by Dunnett’s test. INDO: Indomethacin was used as positive control and was applied at the dose of 1 mg·ear^−1^. LBS: Leaves from plants grow in black soil; SBS: Stems from plants grow in black soil; SRS: Stems from plants grow in red soil.

In the present study, the anti-inflammatory activity at 1 mg·ear^−1^ of the habanero extract by-products obtained by maceration, Soxhlet, and SFE was determined. The results showed that the extracts of leaves of plants grown on black soil extracted by maceration (LBS ME) or supercritical fluid (LBS SFE), and the stems of plants grown on black or red soil extracted by SFE (SBS SFE and SRS SFE, respectively) exhibited statistically significantly higher anti-inflammatory effect in a range from 24.60% to 53.53% compared to the vehicle group. The SRS SFE extract displayed the highest effectiveness (53.53% ± 2.45) at the dose tested (Figure 2). These results confirmed that SFE was an efficient method to obtain bioactive compounds with anti-inflammatory activity. The statistical analysis indicated that the type of extraction, and interaction between type of extraction and type of by-product had a statistically significant effect on the anti-inflammatory effect with a 95% confidence level.

In contrast, none of the samples obtained by Soxhlet extraction showed statistically significant bioactivity. These results were probably due to the high temperature applied in this method which could cause the loss of thermolabile compounds present in the sample [32,33]. Among the methods analyzed, SFE was the most effective confirming the results reported by Eun-Yi et al. [33].

### 2.6. Comparison of the Antioxidant Activity of Extracts Obtained by ME, SOX and SFE

In this section, the antioxidant activity of the extracts of leaves and stems of plants of habanero obtained by three different extraction methods is compared. The statistical analysis indicated that the factors, type of by-products, extraction method, and their interaction displayed statistically significant effects on antioxidant activity with a 95% confidence level. All the samples showed different antioxidant activity, with the stems of plants grown on red soil extracts obtained by maceration being the sample with the highest antioxidant activity (Table 4). For stems of plants grown on both types of soils, maceration was the most efficient method with the highest value of antioxidant activity, while for leaves of plants grown on black soil, Soxhlet was the best method.

It is important to mention that the antioxidant activity of leaves of *C. chinense* var jaguar observed in this study (0.74 mmol Trolox equivalents) was higher than the antioxidant activity of the extract of *C. chinense* var Chichen Itzá obtained by maceration (at 50 °C; 0.05 mmol Trolox equivalent) determined by ABTS [34]. This was consistent with the fact that the antioxidant activity of the samples was depended on the variety, climate, and culture conditions [35].

Unlike the anti-inflammatory effect, where the SFE method was the best, for the antioxidant activity maceration with methanol was the most efficient method. These results underlined the importance of the selection of the extraction method to obtain extracts with different bioactivities useful for different applications [36].

### 2.7. Comparison of Polyphenolic Content of Extracts Obtained by ME, SOX and SFE

A statistically significant difference was observed between the total content of polyphenols detected in the extracts of leaves and peduncles of plants grown on both types of soil regardless to the different extraction techniques. Soxhlet was the method with the highest efficiency in the extraction of polyphenols (3280 ± 15.59 mg·100 g^−1^ DB), and SFE was the method with the lowest efficiency. In addition, Soxhlet was the method that extracted the largest number of compounds. From the extract of leaves of plants grown on black soil, 14 compounds were identified (gallic acid, chlorogenic acid, and hesperidin plus diosmin were not detected in this sample). Supercritical fluid extraction was the method that extracted the lowest number of compounds from the leaves of plants grown on black soil (only myricetin and quercetin plus luteolin were detected).

**Figure 3:**
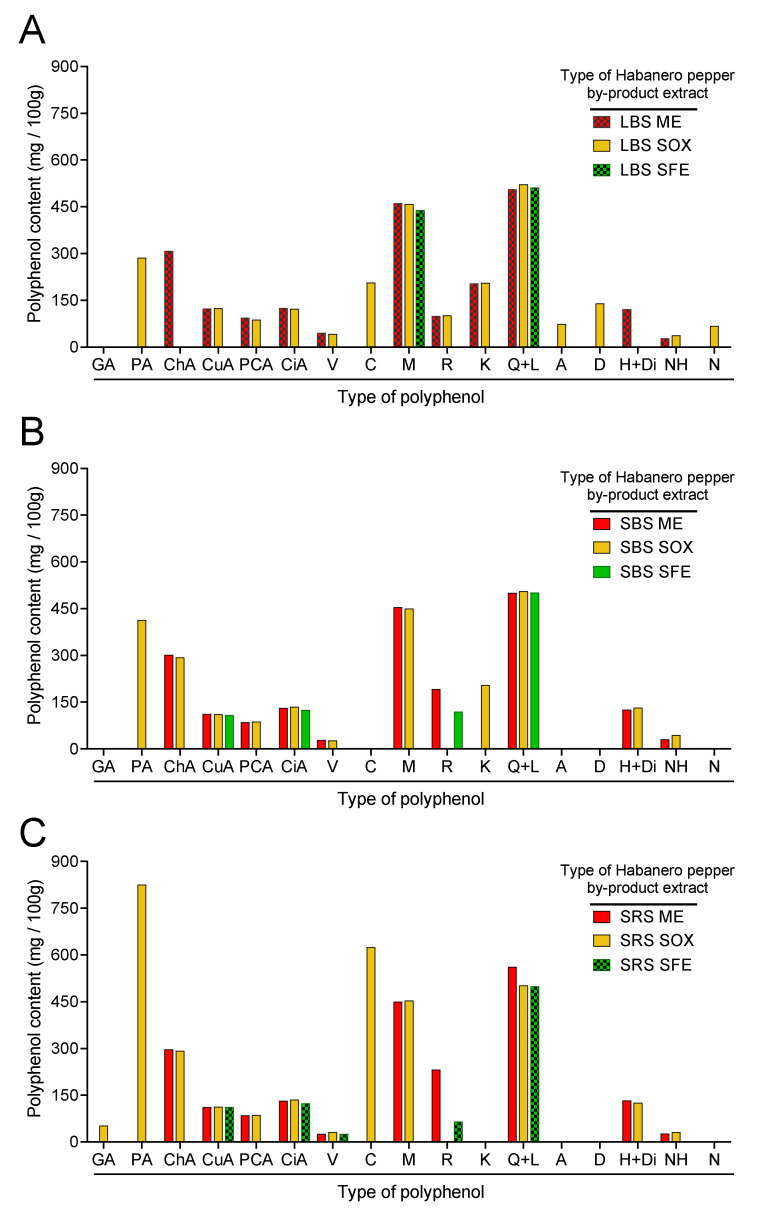
Comparison of the polyphenol content of *C. chinense* by-product extracts obtained by ME, SOX and SFE of leaves of plants grown in black soil (**A**), stems of plants grown in black soil (**B**), stems of plants grown in red soil (**C**) of Yucatán, México. GA: gallic acid; PA: protocatechuic acid; Cha: chlorogenic acid; CuA: coumaric acid; PCA: *p*-coumaric acid; CiA: cinnamic acid; V: vanillin; C: catechin; M: myricetin; R: rutin; K: kaempferol; Q + L: quercetin + luteolin; A: apigenin; D: diosmetin; H + Di: hesperidin + diosmin; NH: neohesperidin; N: naringenin. LBS: leaves from plants grow in black soil; SBS: stems from plants grow in black soil; SRS: stems from plants grow in red soil. ME: maceration extraction with methanol; SOX: Soxhlet extraction with ethanol; SFE: supercritical fluids extraction with CO_2_ + ethanol (5%). Bars with white dots are extracts with anti-inflammatory activity.

The compound detected in the highest amount was protocatechuic acid found in the extract of stems of plants grown on red soils obtained by the Soxhlet method (826.23 ± 7.87 mg ·100 g^−1^ DB). Naringenin, apigenin, and diosmetin were only extracted from leaves of plants grown on black soil by the Soxhlet method (68.03 ± 0.35, 74.63 ± 0.29, and 140.00 ± 30.84 mg·100 g^−1^ DB, respectively), while only the extracts of stems of plants grown on red soil reported gallic acid content (53.03 ± 5.52 mg·100 g^−1^ DB). The extracts of leaves of plants grown on black soil obtained by maceration showed 6 compounds with the highest abundance: chlorogenic acid, coumaric and *p*-coumaric acid, vanillin, myricetin, and kaempferol (308.53 ± 1.53, 123.90 ± 0.46, 46.20 ± 0.72, 94.17 ± 0.06, 461.47 ± 16.66, and 204.07 ± 1.46 mg·100 g^−1^ DB, respectively). There was not a statistically significant difference in kaempferol content between these samples and the extracts of leaves and stems of plants grown on black soil obtained by the Soxhlet method (Figure 3).

The findings reported here agreed with those obtained by Kőszegi et al. [37], who showed a higher content of phenols extracted by Soxhlet with ethanol compared to SFE from the leaves of stinging nettle (*Urtica dioica* L.) with percentages equal to 1.6% and 0.7% pyrogallol equivalent, respectively. According to Tyskiewicz et al. [38], SFE was not an appropriate method to extract the total content of biologically active polyphenolic compounds from *Mentha piperita*, *Origanum vulgare*, *Rosmarinus officinalis*, *Thymus vulgaris*, and mango leaves.

In this study, the analyte recovery rate and the extraction efficiency were affected by the plant matrix characteristics. The nature of target compounds and the relative bonding behavior of the analyte with the extraction solvent were factors affecting the selectivity of the compounds toward the solvents used [20,39]. Therefore, it could be necessary to optimize the conditions of the technologies for the extraction of polyphenols according to the type of raw material.

### 2.8. Correlation between the Anti-Inflammatory Effect or the Antioxidant Activity and Polyphenolic Content of Extracts of C. chinense By-Products Obtained by ME, SOX and SFE

The analysis of the correlation between the polyphenol content and the antioxidant activity of the extracts by-products (Table 5) showed that rutin content had a high positive correlation (r = 0.80, *p* < 0.05). On the other hand, chlorogenic acid, *p*-coumaric acid, quercetin + luteolin, hesperidin + diosmin content had a moderate positive correlation with the antioxidant activity (0.40 ≤ r ≤ 0.60, *p* < 0.05). Between the antioxidant activity and the anti-inflammatory effect, a moderate negative correlation (r = −0.57, *p* < 0.05) was obtained. The analysis of the correlation between the polyphenol content and the anti-inflammatory effect indicated that myricetin content had a high negative correlation (r = −0.70, *p* < 0.05) and chlorogenic acid, *p*-coumaric acid, quercetin + luteolin, hesperidin + diosmin, neohesperidin had a moderate negative correlation with the anti-inflammatory activity (−0.40 ≤ r ≤ −0.69, *p* < 0.05).

The negative correlation suggested that the polyphenols present in the samples reported an antagonistic effect between the anti-inflammatory activity and the antioxidant activity. This result could be related to the presence of other bioactive compounds in the extracts such as terpenoids, or to a synergism between the compounds [40,41]. Grigore et al. [42] tested *V. phlomoides* extract without observing any anti-inflammatory activity. They mentioned that mullein polyphenols contained in the extract played an important role in exerting the antioxidant effect but a weak influence as anti-inflammatory agents due to the high content of iridoids and phenylethanoids compounds.

Therefore, additional studies should be carried out to quantify other compounds with feasible anti-inflammatory activity, as well as synergistic and antagonistic effects between the several bioactive compounds present in the extracts.

### 2.9. Dose–Response Curves of the Extracts That Exhibited Anti-Inflammatory Activity

**Figure 4:**
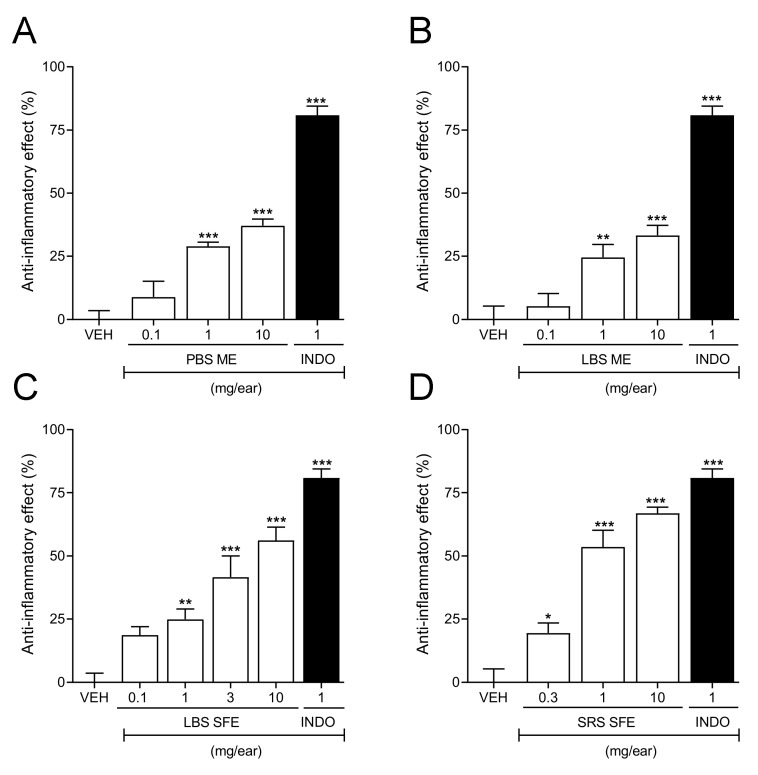
(**A**–**D**) Dose–response curves of the anti-inflammatory effect of the extracts of *C. chinense* by-product extracts obtained by ME and SFE as a function of the amount of extract mg/ear. Each bar represents the mean ± SEM (n = 6–8 ears per dose). * *p* < 0.05, ** *p* < 0.01, *** *p* < 0.001 different from the group treated with the vehicle (VEH), by one-way ANOVA followed by Dunnett’s test. INDO: Indomethacin.

The extracts of the peduncles and leaves of plants grown on black soil obtained by maceration (PBS ME and LBS ME, respectively) and the extracts of leaves of plants grown on black soil and the stems of plants grown on red soil obtained by supercritical fluid extraction (LBS SFE and SRS SFE, respectively) significantly reduced TPA-induced ear edema in a dose-dependent manner. However, PBS ME and LBS ME did not reach an efficacy greater than 50% at the highest dose evaluated (10 mg·ear ^−1^). In contrast, LBS SFE and SRS SFE produced the highest efficacy (61.7 ± 5.3% and 66.1 ± 3.1%) at the same dose (Figure 4). The results indicated that the extracts obtained by SFE were significantly more active than the extracts obtained by maceration.

Consequently, in order to compare the power of each extract, the dose of the extract required to reduce the edema of 30% (ED_30_) was determined. The results showed that the most powerful extract was the SRS SFE (ED_30_ = 0.58 mg·ear^−1^), followed by the LBS obtained by SFE (ED_50_ = 0.64 mg·ear^−1^), while PBS ME and LBS ME displayed ED_30_ values of 2.29 and 3.43 mg·ear^−1^, respectively. It is important to mention that none of the extracts showed greater efficacy compared to the positive control indomethacin. This could be in part due to a synergistic and antagonistic action between some bioactive compounds present in the extracts. Future studies could be interesting to carry out with the aim to identify and isolate the bioactive compounds and test their bioactivities [43].

The results reported here are in agreement with those of Poletto et al. [40], confirming that for extraction of anti-inflammatory compounds, the major inputs came from the SFE method. This method had some advantages compared to conventional techniques. One of the most interesting was related to the tunability of the solvent, given that above the critical conditions of temperature and pressure, the solvent had different values of solubility, diffusivity, and viscosity, thus modifying its extraction power.

## 3. Materials and Methods

### 3.1. Obtaining Extracts

#### 3.1.1. Plant Material

The leaves, stems, and peduncles of *C. chinense* J., variety Jaguar (variety register number CHL-008-101109), considered as by-products of habanero pepper, were obtained from plants cultivated in a greenhouse (15 plants for each type of soil), with controlled irrigation and fertilization conditions [9] at the Centro de Investigación y Asistencia en Tecnología y Diseño del Estado de Jalisco, A.C. (CIATEJ) Subsede Sureste, Mérida, Yucatán, México (Latitude 21°8′1.288″ N and Longitude 89°46′52.26″ W). The crops were planted in polyethylene bags that were filled with 12 kg of two different types of soils characteristic of the Yucatán region: red soil (*Káankab luum*) and black soil (*Box luum*), which were obtained from a local supplier located in Mérida (Yucatán, Mexico). The plants were obtained on harvest number 12 (the last expected harvest, after which the plant is generally wasted), 265 days after the transplantation (DAT) of seedlings (45 days of growth with a minimum height of 19.3 cm and ten true leaves) from the Cutz nursery in Suma de Hidalgo (Yucatán, Mexico), which is characterized by the use of certified seeds. The greenhouse temperature was in the range from 24 to 47 °C and the relative humidity was 91% [15].

#### 3.1.2. Drying of Habanero Pepper By-Products

Dried habanero pepper by-products were obtained according to the methodology reported by Rodríguez-Buenfil et al. [9]. Briefly, the plants were separated into peduncles, leaves, and stems. The stems were chopped using a knife for easy handling. Subsequently, the separated parts were dried in a stainless-steel oven (Novatech, Jalisco, Mexico). The peduncles and stems were dried at 44 °C for 48 h, while the leaves were dried at 44 °C for 240 h to reduce their moisture content to less than 5%. Subsequently, the plant parts were ground and sieved (pore size 500 μm, Sieve # 35, Fisher Scientific, Boston, MA, USA). The obtained powders were stored at −20 °C until analysis.

#### 3.1.3. Maceration Extraction (ME)

Extracts obtained by the maceration method using methanol (ME) were performed according to the methodology reported in Chel-Guerrero et al. [19]. Briefly, 5 g of each sample were weighed and homogenized in 50 mL of methanol HPLC grade (Sigma Aldrich, Naucalpan de Juarez, Mexico), stirred at 160 rpm in a shaking incubator (Novatech, Jalisco, Mexico) for 24 h at room temperature (28 °C). Each extract was filtered with Whatman No. 2 paper. Subsequently, the solvent was evaporated in vacuum at 40 °C, using a rotary evaporator (Buchi, Flawil, Switzerland). All dry extracts were stored at −20 °C until analysis.

#### 3.1.4. Soxhlet Extraction (SOX)

For Soxhlet extraction with ethanol, the methodology described by Paes et al. was used [44]. The method consisted of 150 mL of solvent recycling over 5 g of sample in a Soxhlet apparatus for 3 h extraction at the boiling temperature of the solvent used.

#### 3.1.5. Supercritical Fluid Extraction (SFE)

Extracts by SFE were performed using a supercritical fluid extraction system (Superfluidi s.r.l., Padova, Italy), as described in the work of Ferrentino et al. [18]. The system was equipped with 1 L extractor vessel and two gravimetric separators. The high-pressure vessel contained an extraction basket of 800 mL, closed on both ends with porous stainless steel filter mesh to avoid any possible carryover of samples. The temperature of the extraction and separation vessels was automatically controlled by recirculating the water from two independent thermostated water baths. The CO_2_ was pressurized by a high-pressure diaphragm pump (Lewa LDC—M—9XXV1, Milano, Italy) pre-cooled at 4 °C in order to deliver liquid CO_2_ from a storage cylinder to the extraction vessel efficiently. The system was equipped with a high-pressure tank for CO_2_ storage to be compressed and reused. The extraction was carried out by passing the CO_2_ with ethanol as co-solvent at a concentration of 5% (*w*/*w*), in order to remain in supercritical conditions through the sample. The extract was collected in the separator while the released CO_2_ in the storage tank for further reuse. Briefly, 15 g of powdered samples were extracted at 30 MPa, 45 °C for 2 h. The extraction was performed by loading the sample inside the vessel, and the solvents were flushed through it with a flow rate equal to 2 L·h^−1^. This low flow rate was selected to ensure a long residence time and a prolonged contact between the sample and the solvent. At the end of the extraction time, the extract was collected in amber flasks and weighted using an analytical balance. Moreover, after each extraction, the vessel and the tubes of the system were washed with 25 mL of ethanol to clean the surfaces, minimize the extract losses, and recover all the extracted residues from the *C. chinense* by-products. After the cleaning, the sample was collected in a 250 mL volumetric flask, ethanol was removed by using a rotary evaporator and merged with the extract collected in the amber flask.

#### 3.1.6. Extraction of Polyphenols

The extraction of polyphenols was performed according to the methodology described by Oney-Montalvo et al. [15]. Briefly, a quantity of 0.5 g of each by-product of habanero pepper was mixed with 2.5 mL of solvent, which was composed of methanol:water (80:20) in 15 mL Falcon tubes. The mixture was sonicated at 42 kHz for 30 min at 28 °C (Marshall scientific, Danbury, CT, USA) and then centrifuged at 4816× *g* and 4 °C for 30 min (Centrifuge Mega Fuge 40 R, Thermo scientific, Langenselbold, Germany). The supernatant was collected, and then the centrifugation and recovery of the supernatant were repeated. Finally, the samples were filtered through a 0.2 μm PTFE filter and deposited in amber vials for analysis by UPLC.

### 3.2. Anti-Inflammatory Activity and Dose–Response Curve

#### 3.2.1. Animals

The model of TPA-induced ear inflammation was carried out in strain of female albino mice (CD1 mice), 8–9 weeks old, 30–35 g of body weight. All animals were obtained from our breeding facilities and housed in a temperature-controlled facility (21 ± 2 °C) with a 12/12 h light-dark cycle. Mice had free access to water and standard rodent chow. All experimental procedures were carried out according to the Mexican Official Norm of Animal Care and Handing [45] and the international guidelines on ethical standards for investigation in animals. Moreover, ethics approval for the protocol was obtained from the Institutional Animal Care and Use Committee (No. 0001/2019). Every effort was made to minimize the number of animals used.

#### 3.2.2. TPA-Induced Ear Edema

Ear inflammation was induced following a modified method previously described [46,47]. Under general anesthesia induced by intraperitoneal sodium pentobarbital (63 mg·kg^−1^), TPA dissolved in absolute ethanol (0.25 μg·µL^−1^) was applied in 5 μL volumes to both the inner and outer surfaces of the mouse ear (10 μL·ear^−1^). Five min after TPA application, 20 µL of vehicle (ethanol), the corresponding doses of each habanero pepper extract by-product (0.1–10 μg·ear^−1^, n = 6–8 ears per group) or the positive control (Indomethacin 1 mg·ear^−1^, n = 6–8 ears per group) were applied topically in the same way as TPA. Four hours after TPA application, mice were sacrificed by cervical dislocation and ear biopsies were taken, using a punch of 6 mm of diameter and weighed. Additional biopsies were taken from non-inflamed ear serving as negative control. The degree of edema was indicated by the increase of weight of the ear biopsy treated over the weight of the negative control ear biopsy. Then, % inhibition of ear edema was calculated as follows:

% inhibition of ear edema = 100 − ([(C − E)/C] × 100)(1)

where C is the weight of the ear biopsy treated with vehicle +TPA, and E is the weight of the ear biopsy treated with the corresponding dose of each extract or positive control + TPA. The data of % inhibition of ear edema were used to calculate the percentage of anti-inflammatory effect. The dose–response curves (DRC) were built plotting the percentage of anti-inflammatory effect against the log dose. Linear regression analysis of the log DRC was used to calculate the ED_30_ of each extract.

### 3.3. Antioxidant Activity (ABTS)

The antioxidant activity of the extracts was performed according to the Antioxidant Assay Kit manufacturer’s instructions (Sigma-Aldrich, Naucalpan de Juarez, Mexico) [48]. Briefly, the ABTS substrate working solution was prepared by adding 25 µL of 3% hydrogen peroxide solution to 10 mL of ABTS substrate solution. The samples were prepared by mixing 20 µL of the different habanero by-product extracts obtained by ME, SFE and SOX. The samples of peduncles black and red soils and of stems of black and red soils, were previously diluted 1:25 with methanol with 40 µL of myoglobin working solution and 300 µL of ABTS substrate working solution, allowed to stand for 5 min at room temperature. Then, 200 µL of stop solution was added to stop the reaction and the mixture was allowed to stand at room temperature for one hour. Finally, the absorbance of the samples was read at a wavelength of 405 nm using a spectrophotometer (Jenway, Staffordshire, UK). Trolox was used at different concentrations (0.045, 0.105, 0.21, 0.42 and 0.84 mM) as a standard for the calibration curve and to determine the antioxidant activity of the analyzed samples, expressed in mmol Trolox equivalents·mg^−1^ D.E. (dry extract). Assays were performed in duplicate.

### 3.4. Polyphenolic Determination by UPLC-DAD

Phytochemical quantification was conducted using an UPLC Acquity H Class (Waters, Milford, MA, USA) with a diode array detector (DAD). The column was an Acquity UPLC HSS C18 (100 A°, 1.8 μm, 2.1 mm × 50 mm) (Waters, Milford, MA, USA). Validation of the chromatographic method was carried in accordance with the Eurachem Guide: The Fitness for Purpose of Analytical Methods—A Laboratory Guide to Method Validation and Related Topics [49] reported in a previous work conducted by Chel-Guerrero et al. [50]. Supplementary Materials Table S1 contains the validation values, as well as the chromatographic parameters of the method.

#### Determination of Polyphenolic Content

The quantification of polyphenols by UPLC-DAD was performed using the following conditions: flow speed of 0.5 mL·min^−1^ with a column temperature set at 45 °C and an injection volume of 2 μL. The measurement was performed using the DAD at 280 nm. The polyphenols were separated using acetic acid (0.2%) as solvent A and acetonitrile with acetic acid (0.1%) as solvent B. The elution conditions were as follows: 0–10 min from 1% B to 30% B; 10–12 min 30% B; and 12–15 min from 30% B to 1% B.

The quantification of the polyphenols was performed using two external calibration curves prepared with 17 standards of polyphenols obtained from Sigma Aldrich^®^. The first curve was conformed with: gallic acid, protocatechuic acid, chlorogenic acid, coumaric acid, cinnamic acid, catechin, rutin, kaempferol, quercetin, and luteolin. Meanwhile, the second curve was conformed with: *p*-coumaric acid, vanillin, myricetin, apigenin diosmetin, hesperidin, diosmin, neohesperidin, and naringenin. In both cases, stock solutions (1 mg mL^−1^) of all standards were first prepared. Next, the calibration curves were prepared in the range of 1 to 75 μg mL^−1^. The polyphenols were identified in the samples by comparing their retention time with those of the standards. This method was described by Oney-Montalvo et al. [15]. Quercetin and luteolin, and diosmin and hesperidin were determined together, because the peaks coeluted. The chromatograms of the standards as well as some of the samples analyzed can be found in Supplementary Materials Figures S1–S4.

### 3.5. Statistical Analysis

The results of the antioxidant activity and polyphenol content were expressed as the mean ± standard deviation. Statistically significant differences between the groups were calculated using a two-way analysis of variance (ANOVA). The interactions between groups were analyzed using a multifactorial analysis of variance (the factors analyzed were soil type and by-product type and the interactions between them), followed by the Tukey test. A *p* ≤ 0.05 was considered to be statistically significant. Data analysis was performed using the statistical package Statgraphics Centurion 18.1 18-X64. For all analyses, determinations were made in triplicate as independent experiments, except for protocatechuic acid in leaves of plants that were grown in black soil (LBS), chlorogenic acid in leaves of plants grown in red soil (LRS) and stems of plants grown in black and red soil (SBS and SRS), coumaric acid in peduncles and stems of plants grown on red soil (PRS and SRS), ρ-coumaric acid in leaves of plants grown in black soil (LBS) and peduncles of plants grown in black soil and red soil (PBS and PRS), cinnamic acid (SRS), vanillin (LBS, PRS), catechin (SBS, LRS, SRS), myricetin (SRS), apigenin (LBS, PRS and SRS), diosmetin (SBS), rutin (SRS), kaempferol (PBS and SRS), hesperidin + diosmin (SRS), and neohesperidin (SRS), which were performed in duplicate (in absence of more sample). Pearson’s test was used for the correlation studies and their significance.

The data of in vivo experiments were expressed as the mean ± standard error of the mean (SEM, n = 6–8 per group). The dose–response data were analyzed by one-way analysis of variance (ANOVA) using a Dunnett’s test for the post hoc comparisons, *p* values < 0.05 were indicative of statistical significance. All analyses were performed using the Graph Pad Prism software version 8.0.1 (San Francisco, CA, USA).

## 4. Conclusions

Our findings showed that habanero pepper by-products, currently considered as waste, have potential value for the development of anti-inflammatory drugs to be used for pharmaceutical and food applications. The extracts of leaves, peduncles, and stems of plants grown on black and red soils from Yucatán, México displayed in vivo anti-inflammatory effect and in vitro antioxidant activity. The magnitude of the effect and the activity were affected by the type of extraction and soil used. The supercritical fluid extraction showed good results to obtain compounds with anti-inflammatory activity. Maceration and Soxhlet techniques showed the best results for extracting antioxidants compounds and polyphenols being the stems of plants grown on red soil the most active sample. Protocatechuic acid was the compound found in the highest amount. In conclusion, the results indicated the importance of the selection of the extraction technique to obtain compounds useful for different applications.

## Figures and Tables

**Table 1:** Antioxidant activity of methanolic extracts of habanero pepper by-products of plants grown on black and red soils obtained by maceration.

Sample	Type of Soil	Antioxidant Activity (ABTS) Trolox Equivalent (mM) ^A^
Peduncles	Black soil	2.29 ± 0.0039 ^d^
Leaves	Black soil	0.74 ± 0.0007 ^f^
Stems	Black soil	2.56 ± 0.0026 ^c^
Peduncles	Red soil	2.57 ± 0.0065 ^b^
Leaves	Red soil	0.83 ± 0.0007 ^e^
Stems	Red soil	2.80 ± 0.0052 ^a^

^A^ mmol Trolox equivalent per mg DE. ^a–f^ Different superscript letters in the same row indicates significant differences (*p* ≤ 0.05).

**Table 2:** Polyphenol content of habanero pepper by-product extracts of plants grown on black or red soils of Yucatán, México obtained by maceration.

Polyphenols	Content (mg 100 g^−1^ DB) ^A^					
	Black Soil			Red Soil		
	Peduncles	Leaves	Stems	Peduncles	Leaves	Stems
Chlorogenic acid	292.70 ± 0.75 ^d^	308.53 ± 1.53 ^a^	302.50 ± 3.41 ^b^	293.93 ± 1.10 ^d^	311.13 ± 0.50 ^a^	297.80 ± 0.87 ^c^
Coumaric acid	137.20 ± 1.73 ^b^	123.90 ± 0.46 ^c^	113.07 ± 0.23 ^d^	141.67 ± 0.06 ^a^	137.77 ± 0.25 ^b^	112.10 ± 0.56 ^d^
*p*-coumaric acid	99.60 ± 0.30 ^b^	94.17 ± 0.06 ^c^	86.47 ± 0.06 ^d^	105.30 ± 0.44 ^a^	95.27 ± 2.29 ^c^	86.47 ± 0.06 ^d^
Cinnamic acid	132.03 ± 1.63 ^a^	125.93 ± 1.68 ^c^	131.70 ± 0.20 ^a^	133.23 ± 0.21 ^a^	127.90 ± 0.44 ^b^	133.07 ± 0.67 ^a^
Vanillin	64.43 ± 0.21 ^b^	46.20 ± 0.72 ^d^	29.27 ±0.35 ^e^	71.63 ± 0.31 ^a^	61.47 ± 1.98 ^c^	26.53 ± 0.64 ^f^
Catechin	0.00 ± 0.00 ^b^	0.00 ± 0.00 ^b^	0.00 ± 0.00 ^b^	142.90 ± 0.99 ^a,^*	0.00 ± 0.00 ^b^	0.00 ± 0.00 ^b^
Myricetin	465.13 ± 3.27 ^b^	461.47 ± 16.66 ^b^	454.93 ± 17.12 ^b^	457.70 ± 1.35 ^b^	489.33 ± 16.57 ^a^	450.10 ± 1.55 ^b^
Diosmetin	0.00 ± 0.00 ^b^	0.00 ± 0.00 ^b^	0.00 ± 0.00 ^b^	0.00 ± 0.00 ^b^	123.10 ± 0.00 ^a,^*	0.00 ± 0.00 ^b^
Rutin	239.30 ± 8.88 ^b^	99.43 ± 2.25 ^e^	192.23 ± 2.34 ^c^	265.13 ± 1.63 ^a^	116.15 ± 2.76 ^d^	232.00 ± 9.85 ^b^
Kaempferol	208.33 ± 4.05 ^a^	204.07 ± 1.46 ^b^	0.00 ± 0.00 ^c^	203.67 ± 2.34 ^b^	204.70 ± 2.48 ^a,b^	0.00 ± 0.00 ^c^
Quercetin + Luteolin **	531.37 ± 0.61 ^b^	506.77 ± 9.47 ^d^	500.77 ± 1.87 ^d^	519.00 ± 0.82 ^c^	505.30 ± 0.79 ^d^	562.10 ± 1.47 ^a^
Hesperidin + Diosmin **	187.97 ± 0.49 ^b^	122.57 ± 4.16 ^e^	126.40 ± 0.95 ^e^	204.77 ± 0.81 ^a^	143.33 ± 6.07 ^c^	133.80 ± 1.93 ^d^
Neohesperidin	36.63 ± 1.15 ^a^	28.53 ± 2.40 ^b^	31.57 ± 5.35 ^a,b^	33.10 ± 5.11 ^a,b^	36.23 ± 1.26 ^a^	27.47 ± 0.31 ^b^
Naringenin	67.13 ± 0.15 ^a^	0.00 ± 0.00 ^d^	0.00 ± 0.00 ^d^	65.97 ± 0.15 ^b^	63.23 ± 0.32 ^c^	0.00 ± 0.00 ^d^
Total Content ^B^	2462.83 ± 10.98 ^b^	2121.57 ± 23.97 ^d^	1968.90 ± 14.48 ^e^	2638.00 ± 87.44 ^a^	2414.92 ± 86.65 ^c^	2061.43 ± 10.43 ^d^

^A^ DB: Dry basis; Mean ± SD (n = 3); * mean ± SD (n = 2); ** were determined together because separation of the peaks by UPLC was not clear; ^B^ Expressed as the sum of the individual polyphenol contents in the analyzed samples as mg 100 g^−1^ dry basis; ^a–f^ Different superscript letters in the same row indicates statistically significant differences (*p* ≤ 0.05).

**Table 3:** Correlation matrix (Pearson’s correlation coefficients) for the anti-inflammatory effect, antioxidant activity, and polyphenol content of *C. chinense* by-product extracts obtained by maceration.

Compounds or Bioactivity	Anti-Inflammatory Effect (% Inhibition)	Antioxidant Activity (ABTS) ^1^
Chlorogenic acid	0.11	−0.83
Coumaric acid	0.24	−0.28
*p*-Coumaric acid	0.19	−0.11
Cinnamic acid	−0.52	0.97
Vanillin	0.26	− 0.26
Catechin	−0.49	0.32
Myricetin	0.29	−0.72
Rutin	−0.38	0.92
Kaempferol	0.54	−0.59
Quercetin + Luteolin	−0.14	0.56
Diosmetin	−0.03	−0.60
Hesperidin + Diosmin	−0.01	0.37
Neohesperidin	0.28	−0.14
Naringenin	0.13	−0.06
Antioxidant activity (ABTS) ^1^	−0.51	1.00
% Anti-inflammatory effect	1.00	

^1^ mmol Trolox equivalent (mM) per mg DE.

**Table 4:** Antioxidant activity of extracts of leaves of plants grown in black soil and stems of *C. chinense* of plants grown on black and red soil obtained by ME, SOX and SFE.

Sample	Type of Soil	Type of Extraction	Trolox Equivalent (mM) ^A^
Leaves	Black soil	ME	0.74 ± 0.0007 ^g^
Leaves	Black soil	SOX	0.80 ± 0.002 ^d^
Leaves	Black soil	SFE	0.75 ± 0.001 ^f^
Stems	Black soil	ME	2.56 ± 0.0026 ^b^
Stems	Black soil	SOX	0.83 ± 0.0013 ^c^
Stems	Black soil	SFE	0.58 ± 0.0023 ^i^
Stems	Red soil	ME	2.80 ± 0.0052 ^a^
Stems	Red soil	SOX	0.79 ± 0.0016 ^e^
Stems	Red soil	SFE	0.73 ± 0.0016 ^h^

^A^ mmol Trolox equivalent per mg DE. ^a–i^ Different superscript letters in the same row indicates significant differences (*p* ≤ 0.05).

**Table 5:** Correlation matrix (Pearson correlation coefficient) performed on data obtained from extracts of leaves of *C. chinense* grown on black soil and stems of plants grown on black and red soils obtained by ME, SOX and SFE.

Compound	Anti-Inflammatory Effect (% Inhibition)	Antioxidant Activity (ABTS) ^1^
Gallic Acid	−0.32	−0.17
Protocatechuic Acid	−0.37	−0.29
Chlorogenic Acid	−0.63	0.48
Coumaric Acid	−0.13	0.15
*p*-Coumaric Acid	−0.69	0.40
Cinnamic Acid	−0.22	0.23
Vanillin	−0.14	0.13
Catechin	−0.33	−0.22
Myricetin	−0.70	0.34
Rutin	−0.31	0.80
Kaempferol	−0.01	−0.34
Quercetin + Luteolin	−0.40	0.60
Apigenin	−0.03	−0.17
Diosmetin	−0.03	−0.17
Hesperidin + Diosmin	−0.67	0.52
Neohesperidin	−0.65	0.28
Naringenin	−0.03	−0.17
Antioxidant activity (ABTS) ^1^	−0.57	1.00
Anti-inflammatory effect (% Inhibition)	1.00	

^1^ mmol Trolox equivalent (mM).

## Data Availability

Not applicable.

## Supplementary Materials

The following are available online, Figure S1: Chromatograms of: (a) Blank, MeOH:H2O (80:20), (b) Mix of standards at 75 µg·mL^−1^ (gallic acid, protocatechuic acid, catechin, chlorogenic acid, coumaric acid, cinnamic acid, rutin, quercetin + luteolin and kaempferol), (c) Mix of standards at 75 µg·mL^−1^ (vanillin, ρ-coumaric, myricetin, hesperidin + diosmin, neohesperidin, naringenin, apigenin and diosmetin), Figure S2: Chromatogram of: (a) Peduncles extracts obtained from habanero pepper plants grown in red soil, (b) Leaves extracts obtained from habanero pepper plants grown in red soil, (c) Stems extracts obtained from habanero pepper plants grown in red soil. All extracts were obtained by maceration with methanol, Figure S3: Chromatogram of: (a) Stems from plants grow in red soil by maceration extraction with methanol, (b) Stems from plants grow in red soil by Soxhlet extraction with ethanol, (c) Stems from plants grow in red soil by supercritical fluids extraction with CO_2_ + ethanol (5 %), Figure S4: Chromatogram of: (a) Stems from plants grow in black soil by Soxhlet extraction with ethanol, (b) Stems from plants grow in red soil by Soxhlet extraction with ethanol, Table S1: Data from the chromatographic parameters and instrumental validation of the chromatographic method developed for the quantification of polyphenols in Habanero pepper by-products.
