# Supplementary material for: In Vivo Anti-Inflammatory Effect, Antioxidant Activity, and Polyphenolic Content of Extracts from Capsicum chinense By-Products
Source: Molecules. 2022 Feb 16;27(4):1323. doi: 10.3390/molecules27041323 (PMC8880488; doi:10.3390/molecules27041323)
Supplement: Supplementary file 1 [file molecules-27-01323-s001.zip › molecules-1577650-supplementary.pdf]

Supplementary material

Table S1. Data from the chromatographic parameters and instrumental validation of the chromatographic method developed for the quantification of polyphenols in Habanero pepper by-products.

**Polyphenols**	**Chromatographic parameters**			**Validation of the chromatographic method**				
	K′	***N***	***H* (μm)**	**LOD (μg/mL)**	**LOQ (μg/mL)**	**Linearity (r^2)**	**RSD % (Intraday)**	**RSD % (Interday)**
Gallic acid	0.68	64	781.25	0.05	0.18	0.9996	1.16	23.64
Protocatechuic acid	3.87	754	66.33	0.02	0.88	0.9996	1.49	6.43
Chlorogenic acid	11.42	16469	3.04	0.06	0.95	0.9990	1.17	2.11
Coumaric acid	14.00	17651	2.83	0.07	0.82	0.9995	0.11	17.47
Cinnamic acid	16.26	17889	2.80	0.11	0.73	0.9997	0.08	9.82
Vanilim	13.52	22500	2.22	0.03	0.94	0.9992	0.23	2.83
Catechin	11.10	27778	1.80	0.02	0.46	0.9997	3.10	19.59
Myricetin	21.29	63138	0.79	0.04	0.55	0.9950	1.00	14.12
Apigenin	29.39	84011	0.60	0.44	0.67	0.9997	0.90	3.27
Diosmetin	30.71	127774	0.39	0.25	0.84	0.9996	0.73	2.43
Rutin	17.55	82656	0.60	0.03	0.77	0.9991	0.58	4.42
Kaempferol	27.77	24065	2.08	0.02	0.25	0.9999	0.54	7.84
Quercetin + Luteolin	24.23	22187	2.25	0.06	0.97	0.9993	0.60	13.10
Hesperidin + Diosmetin	22.94	72802	0.69	0.01	0.25	0.9992	0.71	0.96
Neohesperidin	23.68	93636	0.53	0.03	0.58	0.9986	0.86	1.28
Naringenin	28.52	68345	0.73	0.15	0.51	0.9997	0.67	1.04

Note: *K′*= Capacity factor; *N* = Number of theoretical plates; *H* = Plate height; LOD = Limit of detection; LOQ = Limit of quantification; RSD = Relative standard deviation.

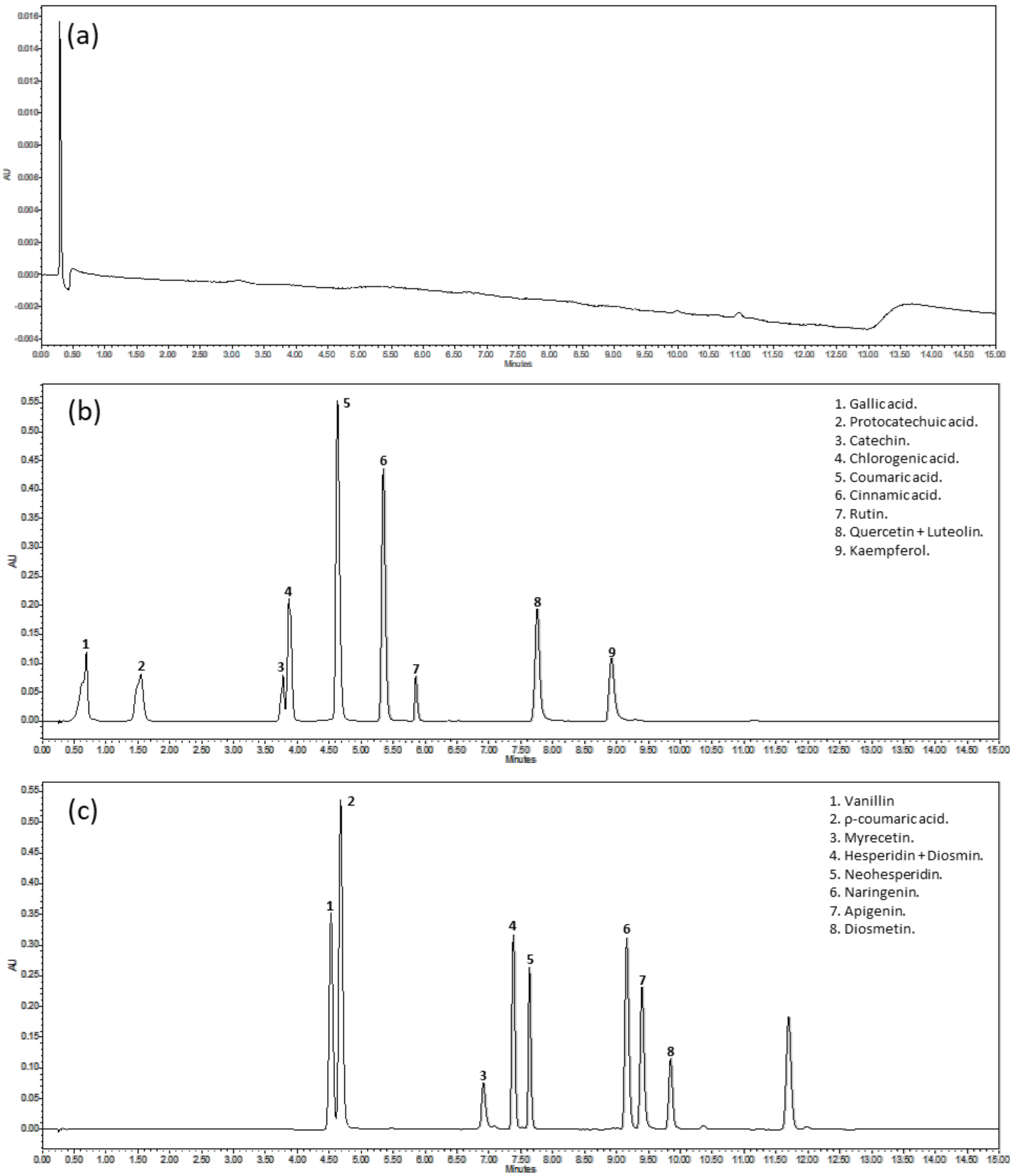

Figure S1. Chromatograms of: (a) Blank, MeOH:H_2O (80:20). (b) Mix of standards at 75 μg mL^{-1} (gallic acid, protocatechuic acid, catechin, chlorogenic acid, coumaric acid, cinnamic acid, rutin, quercetin + luteolin and kaempferol). (c) Mix of standards at 75 μg mL^{-1} (vanillin, ϱ-coumaric, myricetin, hesperidin + diosmin, neohesperidin, naringenin, apigenin and diosmetin).

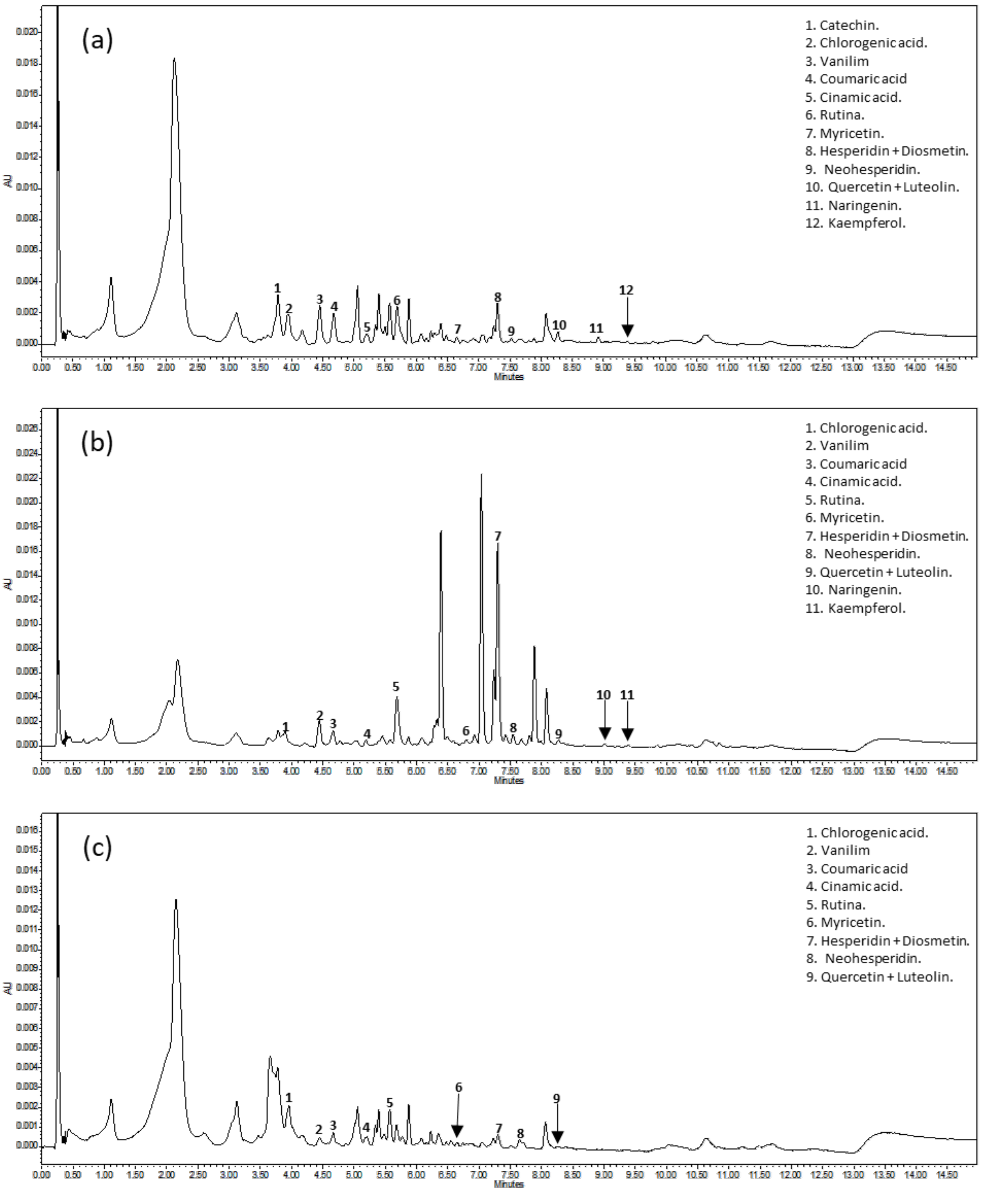

Figure S2. Chromatogram of: (a) Peduncles extracts obtained from habanero pepper plants grown in red soil. (b) Leaves extracts obtained from habanero pepper plants grown in red soil. (c) Stems extracts obtained from habanero pepper plants grown in red soil. All extracts were obtained by maceration with methanol.

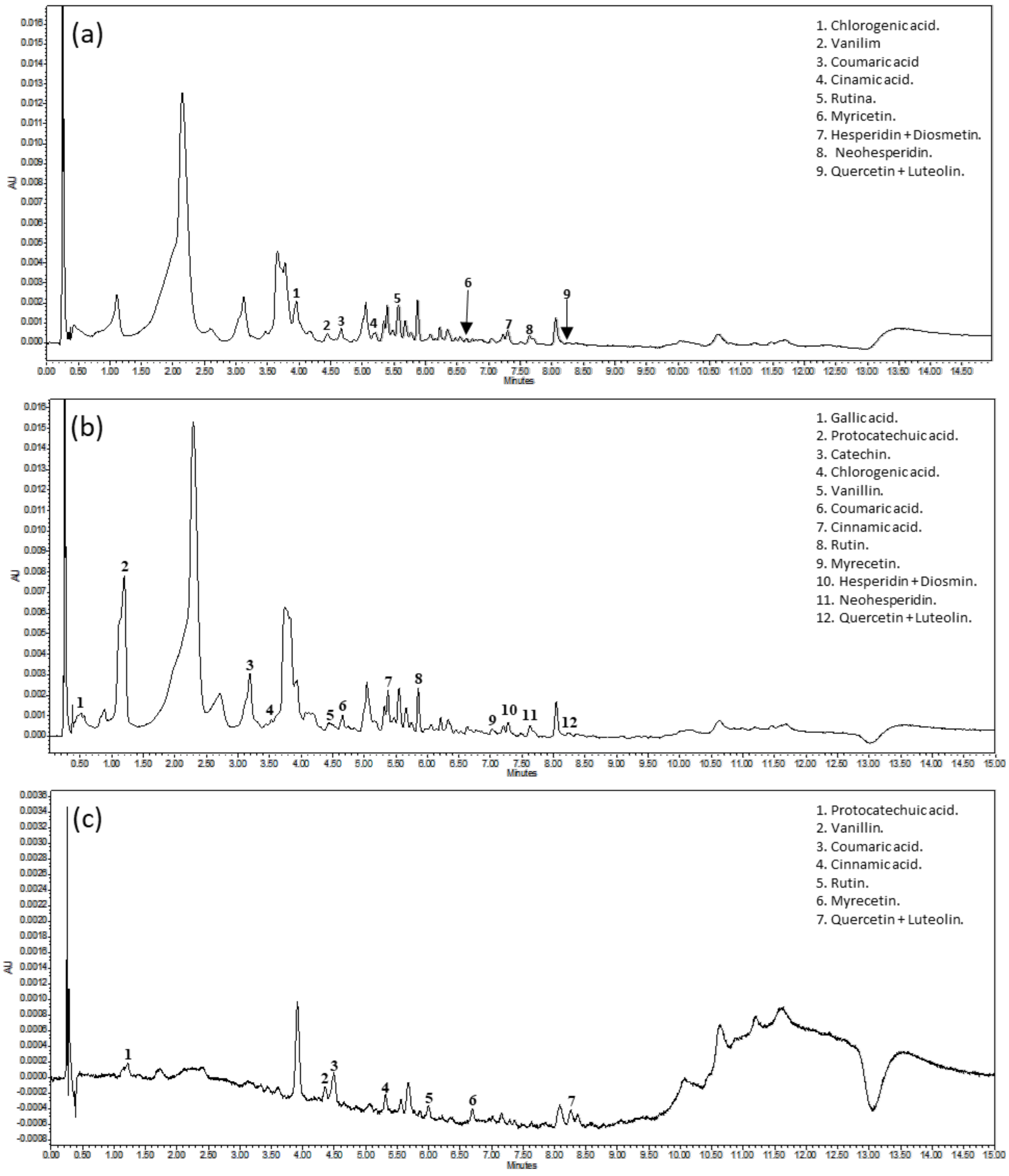

Figure S3. Chromatogram of: (a) Stems from plants grow in red soil by maceration extraction with methanol. (b) Stems from plants grow in red soil by Soxhlet extraction with ethanol. (c) Stems from plants grow in red soil by supercritical fluids extraction with CO2 + ethanol (5 %).

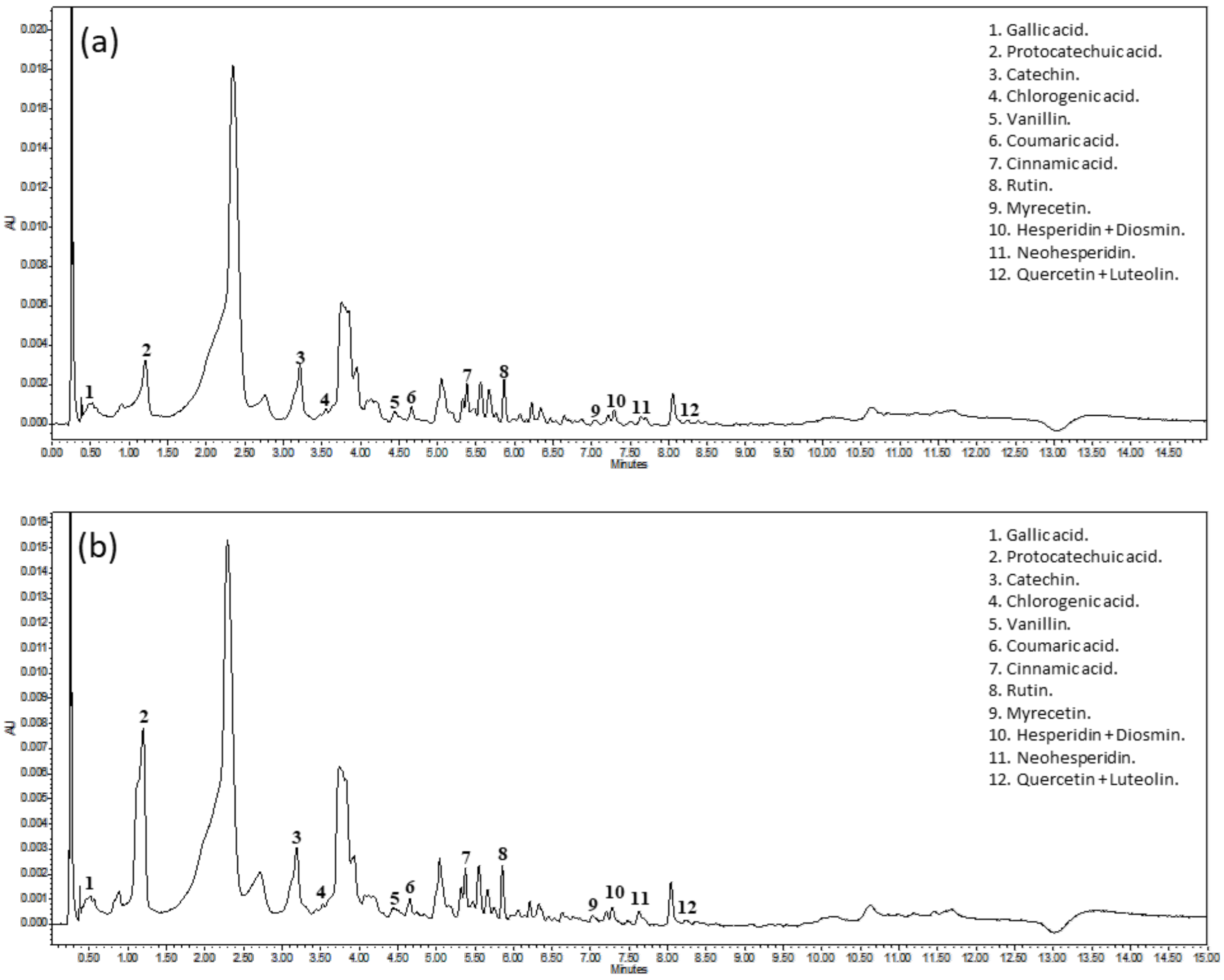

Figure S4. Chromatogram of: (a) Stems from plants grow in black soil by Soxhlet extraction with ethanol. (b) Stems from plants grow in red soil by Soxhlet extraction with ethanol.
